# Supplementary figures and images for: Analytical performance of 17 commercially available point-of-care tests for CRP to support patient management at lower levels of the health system
Source: PLoS One. 2023 Jan 20;18(1):e0267516. doi: 10.1371/journal.pone.0267516 (PMC9858008; doi:10.1371/journal.pone.0267516)

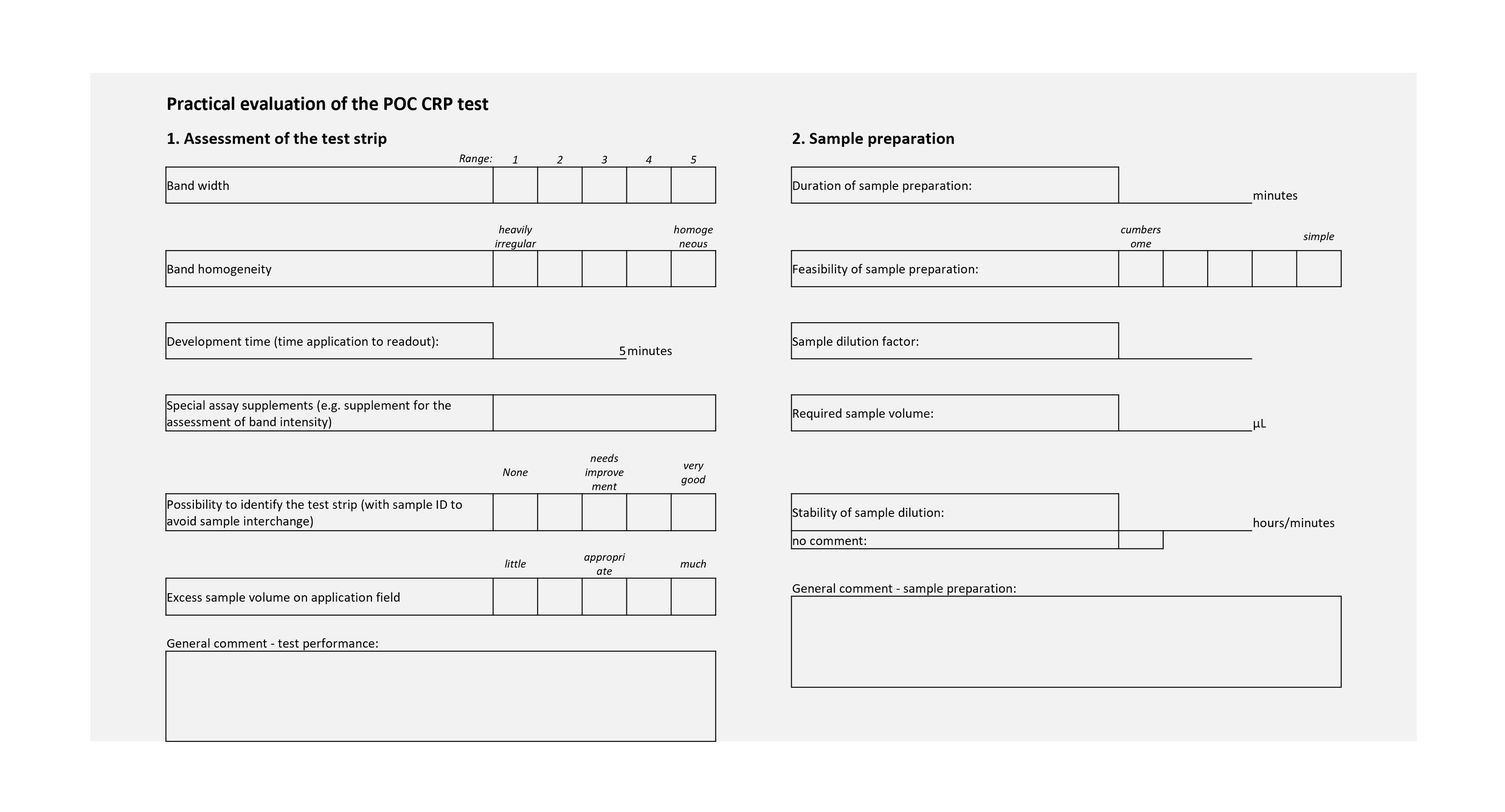

Supplement: S1 Fig — (TIF) [file pone.0267516.s001.tif]

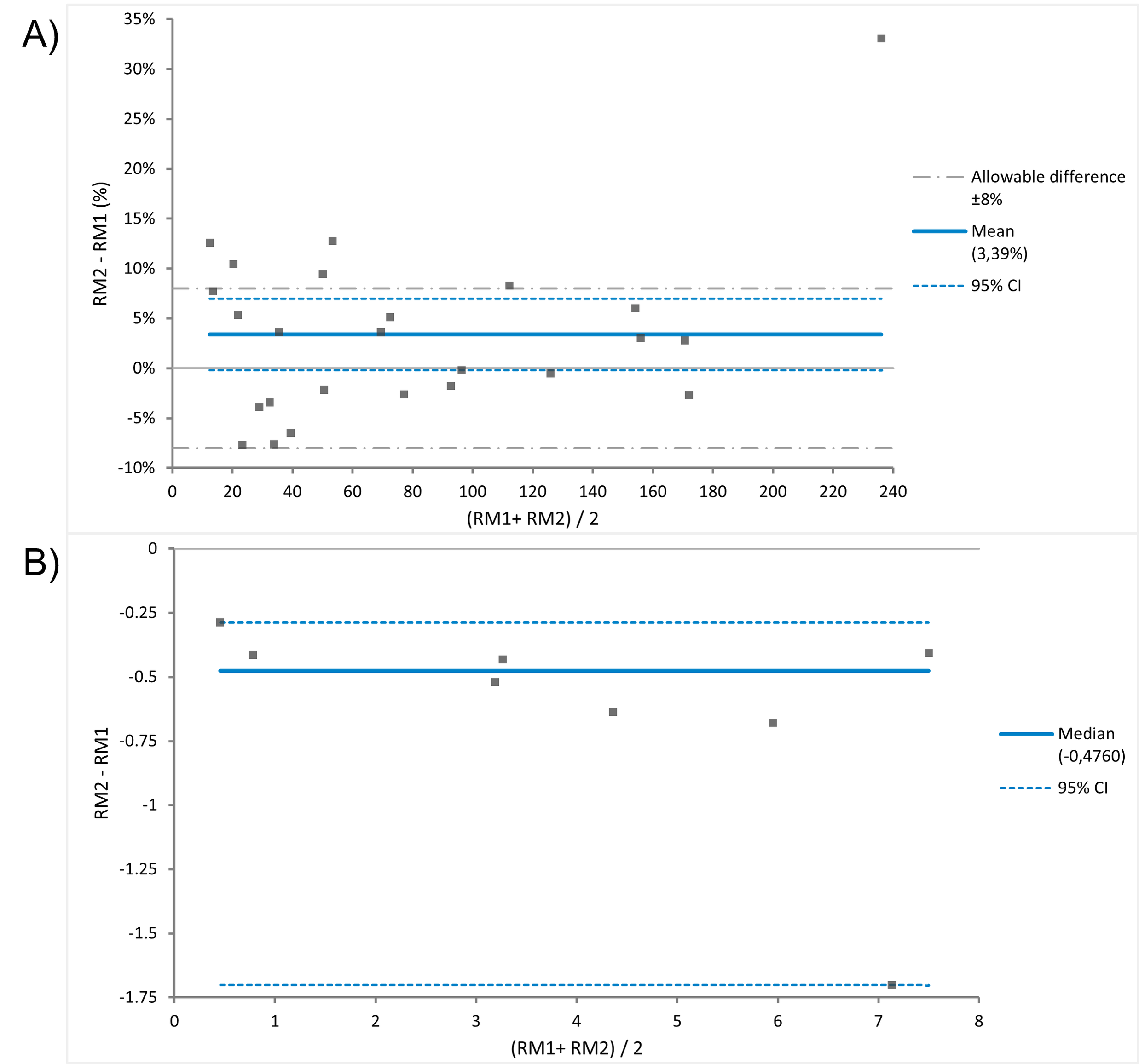

Supplement: S2 Fig — A) excluding values <10 mg/L; B) values from 0 to 10 mg/L. Gray solid line: bias, grey dotted line: 90% confidence interval band. Gray dash line: allowable range. Symbol star: outlier not included in analysis. (TIF) [file pone.0267516.s002.tif]

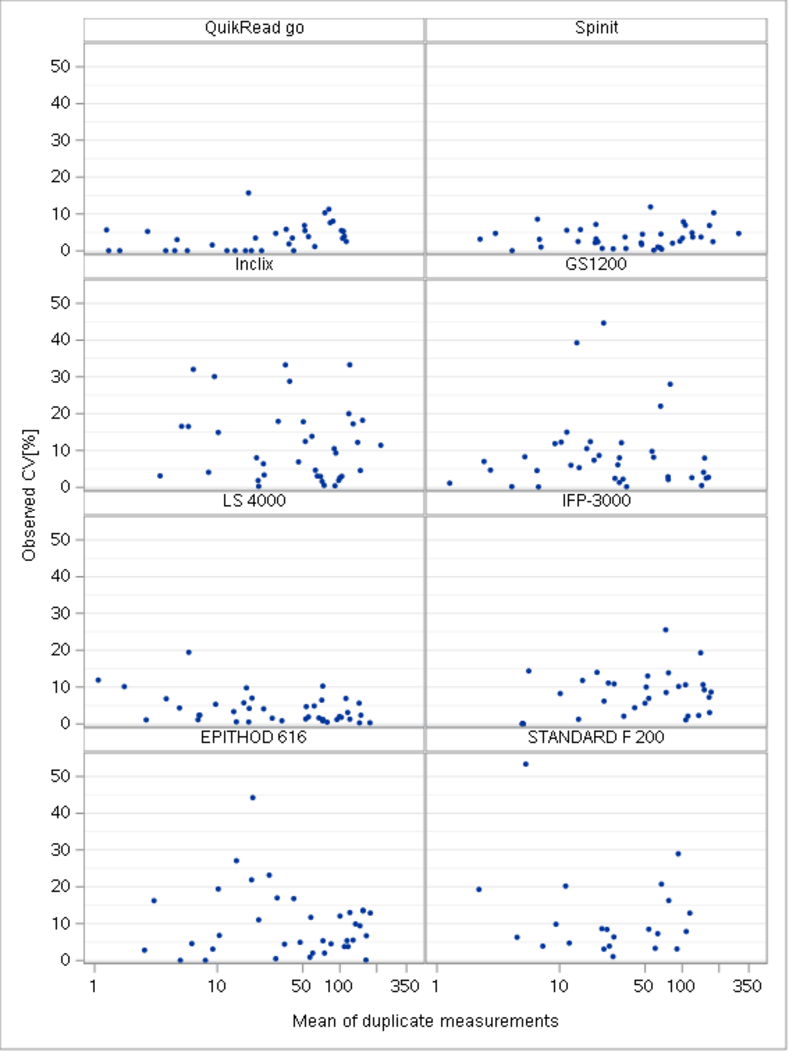

Supplement: S3 Fig — (TIF) [file pone.0267516.s003.tif]
